# Osteoporosis: A Narrative Review

**DOI:** 10.7759/cureus.43031

**Published:** 2023-08-06

**Authors:** Azizi Sheik Ali

**Affiliations:** 1 Department of Respiratory Medicine, University of Exeter, Exeter, GBR

**Keywords:** bone densities, fragility fractures, bone mineral density, quality of life, keywords: osteoporosis

## Abstract

Osteoporosis is a disease of global concern, with significant implications for mortality, morbidity, strain on national health resources, and the negative impact on the quality of life associated with the condition. As we witness a primarily aging population, future predictions indicate that risk factors for osteoporosis will be more prevalent, leading to an increase in the number of individuals suffering from the condition and associated fractures. However, the future of osteoporosis in terms of diagnosis and treatment is optimistic. Understanding of bone quality and examination of it has improved with the onset of magnetic resonance imaging (MRI) and other imaging techniques such as micro-computer tomography. Innovative therapies specifically targeting osteoporotic bone metabolism on a microscopic level hold promise. This narrative review provides details on the background, prognosis, and future treatment strategies of osteoporosis.

## Introduction and background

Osteoporosis is widely considered to be a silent condition. It is defined as having a bone mineral density (BMD) with a standard deviation difference equal to or less than 2.5 from ordinary high levels for fit young adults [1,2]. Its clinical significance can be attributed mainly to the fragility fractures resulting from the onset of this asymptomatic disease, with marked increases in the risk of fractures in the proximal femur, distal radius, and vertebral compression being the most prevalent. Mortality following proximal femur fracture is also considerably raised, by a substantial 15-20%. Remarkably, 70% of vertebral fractures do not come to medical attention [3], primarily due to the generic nature of presenting symptoms such as back pain and loss of height, which are often dismissed as a predestined part of ageing.

Not only does the condition result in hundreds of thousands of fractures every year, but it also imposes a significant cost to the NHS, amounting to over £940 million. This leads to a 20% requirement of orthopaedic bed occupancy and brings about obvious disability and distress to osteoporotic patients [4]. Therefore, the clinical significance of the condition is paramount.

Fractures following the onset of osteoporosis are increasing on an international level in terms of both number and proportion. This change is attributed mainly to the associated decline in physical activity (resulting in increased frailty) and an ageing population [5]. The future of osteoporosis in terms of diagnosis is hopeful: assessments of not just BMD but also bone quality, a measure of bone microarchitecture, turnover, mineralization, and cellularity, will help provide a greater degree of understanding of fracture risk. New therapeutic options that specifically target molecules of bone metabolism have also shed light on greater improvements in our diagnosis and treatment of osteoporosis over the last ten years [6]. This narrative review details the background, prognosis, and current and future treatment strategies of osteoporosis.

## Review

Incidence

Osteoporosis is a key public health threat, affecting approximately 40% of women over 70 years in the United Kingdom [4]. In the United States, 55% of American citizens over the age of 50 are osteoporotic, with a significant proportion (80%) of these being female [7]. Having low BMD is understood to be a central risk factor for developing fractures, most notably proximal femur fractures. As such, a high number of women suffer from osteoporosis. These figures point to something quite alarming: more than 33% of all adult women will eventually suffer from an osteoporotic fracture [8].

Proximal femur (hip) fractures are regarded as the most critical of all osteoporotic fractures since they are coupled with significant morbidity, mortality, and strain on national healthcare [9]. Global studies conducted from 1930 to 1980 have shown annual increases in hip fracture prevalence over the decades, with this rise being even more pronounced among women. The mortality that arises from hip fractures is vast, estimated to be in the region of 15-20% following the onset of fracture, which is slightly lower for vertebral and distal forearm fractures.

Currently, over 50% of all hip fractures occur in North America and Europe, but as the elderly populations of particularly Latin America and Asia increase quite drastically, by 2050, it is predicted that this proportion will fall to 25% in North America and Europe [10].

Prognosis

It has been suggested that 95% of all osteoporotic patients who have suffered hip fractures will require surgery [11]. Worse still, mortality can result from the risk of infection during surgery, further complicating matters. Vertebral fractures have many consequences as well. Kyphosis is a condition where compression of the vertebrae results in excess curvature of the spine and, thereby, loss of height, which can also impair breathing. Chronic pain is yet another result of these fractures.

The health-related quality of life of patients following osteoporotic fractures is often poor, leading to disability and a lack of self-supporting living. Indeed, only half of the patients will return to complete independence following a fracture, and there is an increase of roughly nine consultations with their GP the year after. It has been estimated that 20-50% of patients following a hip fracture will require long-term nursing home care, which is associated with a decreased quality of life [12]. Moreover, after a patient has had one previous vertebral compression fracture, the risk of having yet another one escalates by a shocking 500%. Thus, fractures severely impact the quality of life of osteoporotic patients in many respects, making osteoporosis a startling international healthcare problem [13].

US studies have pointed out that this impact on the quality of life is the case regardless of age group after menopause for women. They have called for primary and secondary preventive methods to reduce such high levels of mortality and morbidity and to improve the quality of life for osteoporotic patients before and after fracture [14].

Primary preventive methods mainly tackle the morbidity of osteoporosis by diminishing the number of falls, particularly in post-menopausal women. These methods include introducing weight-bearing exercises in the community and enhancing the diet with supplements to optimize BMD. Patients are encouraged to lead a bone-healthy lifestyle by quitting smoking, reducing alcohol intake, and increasing their level of activity to reduce the risk of fragility fractures and promote bone health [15]. As a large majority of all hip fractures are caused by falls, this preventive approach is crucial in helping to tackle the risk of osteoporotic fractures [16].

The FRAX algorithm (Fracture Risk Assessment Tool) has been developed by the World Health Organization to aid clinicians in examining the comparative risk of fracture. Since osteoporosis is an asymptomatic condition that often only presents post-fracture, it is essential to be able to assess the risk before such a fracture occurs. The FRAX tool helps clinicians determine whether a DXA (dual-energy X-ray absorptiometry) scan is required to measure BMD and further assess the risk of fracture.

Following NICE (National Institute for Health and Care Excellence) guidelines [17], after performing a FRAX assessment on patients at risk of osteoporosis (i.e., women over 65 or men over 75), the next step in management is based on the probability of a major osteoporotic fracture from the FRAX score.

Low Risk of Fragility Fracture

Those below the 10% recommended threshold are not offered drug treatment but rather lifestyle advice such as reducing alcohol intake and smoking.

Intermediate Risk of Fragility Fracture

Those that fall close to but are below the 10% threshold and have risk factors that are underestimated by the FRAX assessment, that is, heavy alcohol intake, are advised to arrange a DXA scan to measure BMD. Drug treatment is offered if the T-score is negative 2.5 or below.

High Risk of Fragility Fracture

Those that are 10% or above the recommended threshold are offered a DXA scan, followed by a BMD measurement, and subsequently a bone-sparing drug treatment if the T-score is negative 2.5 or below. Risk factors are modified where possible, and DXA scans are repeated at an interval that usually falls within two years.

The main pharmacological options for osteoporosis are categorized into antiresorptive and anabolic agents, with the most mainstreamed being the antiresorptive agent bisphosphonate (BP). The fundamental goal of the therapeutic approach is to reduce the risk of osteoporotic fractures.

BP has been used in the management of osteoporosis for decades, primarily because it is relatively cheap and can be administered in different forms: orally (e.g., alendronate) and intravenously (e.g., zoledronic acid) [18]. The mechanism of action of BP is to induce osteoclast apoptosis which in turn reduces bone resorption, preserving bone tissue by preventing an imbalance. BP has a P-C-P bone central to its structure with a high binding affinity towards hydroxyapatite, a key mineral component of bone. This enables BP to be subsequently adsorbed by osteoclasts to inhibit their activity [18]. NICE guidelines suggest If an oral BP is not tolerated or is contraindicated, specialist referral should be considered [17].

Future

Much improvement has been seen in this century in terms of both the diagnosis and treatment of osteoporosis, and this is set to continue as our understanding of the condition improves [6]. The previous notion that BMD measured by a DXA scanner provides a thorough examination of bone is now considered to be not so simple [19]. Bone is a tissue of great complexity, with bone quality being crucial in terms of fracture risk, and thus, the diagnosis of osteoporosis: bone microarchitecture, turnover, mineralization, and cellularity all play a role here. Indeed, the traditionally used DXA scanner has been found to be quite inaccurate, with estimates of BMD deviating from the actual value by a median figure of 35% [19].

Fracture liaison services (FLS) have been effective within the UK in identifying, investigating, and treating patients with fragility fractures. Patients who have experienced a fragility fracture can receive support in onward management to prevent further fractures. However, currently, only 60% of the UK population has access to this service, and half of the service operations were ceased following COVID. The Royal Osteoporosis Society has suggested that opportunities to expand this service, such as nationalization, may bring about a positive change in the well-being of many people [3].

The International Osteoporosis Foundation (IOF) and International Federation of Clinical Chemistry and Laboratory Medicine (IFCC) Working Group suggest that bone turnover markers, such as S-P1NP (serum procollagen type 1 N-terminal propeptide) (bone formation) and s-CTX (serum C-terminal telopeptide) (bone resorption), may independently predict future fracture risk from BMD. This discovery is promising as their inclusion in assessment algorithms, along with further research, may provide us with a greater ability to diagnose and treat the condition [20].

The significance of osteoporosis in terms of the great mortality and morbidity following fractures and the high risk of re-fracture has received increased international focus [6].

Therapeutic options for osteoporosis have also advanced and are set to progress further in the coming years. Emerging medications include inhibitors of Cathepsin K, a prevalent protein protease in osteoclasts involved in bone degradation [21], and romosozumab, which are monoclonal antibodies that inhibit the action of sclerostin, an inhibitor of bone formation, particularly in the elderly skeleton [22]. With further research on these pioneering therapeutic options, they may potentially become mainstream alongside the currently popular BP anti-resorptive medications used to treat osteoporosis.

## Conclusions

It is clear that osteoporosis is a major public health threat with significant morbidity and mortality associated with the condition. It is also highly debilitating too: only half of all patients following hip fractures will be able to resume completely independent lives, requiring much more assistance from clinicians and other healthcare professionals. This puts significant stress on national health services in terms of bed occupancy and healthcare economics. One crucial factor to note here is that osteoporosis is principally an asymptomatic disease, usually diagnosed and treated only after the onset of fragility fractures. Therefore, prevention, as opposed to therapeutic options, is crucial. Dietary supplements and weight-bearing exercises, particularly for postmenopausal women, need to become more widespread to prevent the recent surge in osteoporotic fractures on a global level and the predicted increases yet to be witnessed over the years.

The future of osteoporosis is predicted to be bleak, particularly in terms of morbidity. As the proportion of people in the elderly population bracket increases, and physical activity among many decreases on an international level, the frequency of osteoporotic fractures is set to increase dramatically too. However, recent advancements in the early diagnosis and understanding of bone tissue, coupled with innovative therapeutic options, shed light on improvements in the diagnosis and treatment of the disease.
